# Reasons for non-acceptance of statin therapy by patients at high cardiovascular risk

**DOI:** 10.1038/s41598-025-01930-2

**Published:** 2025-05-16

**Authors:** Minjia Xie, Seth S. Martin, Alexander Turchin

**Affiliations:** 1 Northeastern University School of Pharmacy, Boston, MA USA; 2 Johns Hopkins Medicine, Baltimore, MD USA; 3 Division of Endocrinology, Brigham and Women’s Hospital, 221 Longwood Avenue, Boston, MA 02115 USA; 4 Harvard Medical School, Boston, MA USA

**Keywords:** Cardiology, Dyslipidaemias

## Abstract

Statins are a cornerstone of cardiovascular risk reduction. Nevertheless, non-acceptance of statin therapy recommendations by patients at high cardiovascular risk is common. The reasons for statin non-acceptance have not been well established. We conducted a manual record review of a randomly selected set of patients who did not accept statin therapy recommendations to identify (a) documented reasons for statin non-acceptance and (b) patients’ demographic characteristics, comorbidities and current treatment. We analyzed the relationships between patients’ characteristics and reasons for statin non-acceptance. The most common reasons for statin non-acceptance were preference for lifestyle modifications (51.5%), general aversion to medications (19.1%), polypharmacy burden (17.1%) and fear of adverse reactions (10.9%). Patients taking more medications were more likely to express a concern about polypharmacy burden (OR 1.09; 95% CI 1.005–1.18). Patients who previously had adverse reactions to non-cholesterol lowering medications were more likely to fear adverse reactions to statins (OR 1.13; 95% CI 1.001–1.28). Patients who expressed preference for lifestyle modifications had time to low density lipoprotein cholesterol (LDL-C) < 100 mg/dL similar to patients who did not accept statin therapy for other reasons (1935 vs. 1777 days, *p* = 0.26). Patients’ reasons for non-acceptance of statin therapy are often linked to their past and present medical experience. Appropriately addressing these concerns is important to maximizing cardiovascular risk reduction in individuals who may be reluctant to initiate statin therapy.

## Introduction

Atherosclerotic cardiovascular disease (ASCVD) is the most common cause of death in the U.S. and worldwide^1^. Elevated low density lipoprotein cholesterol (LDL-C) levels are a common risk factor for ASCVD^2^. Hydroxymethylglutaryl-CoA (HMG-CoA) reductase inhibitors, or statins, are the cornerstone of treatment of hypercholesterolemia^3^. Statins reduce the incidence of major cardiovascular events, in particular in patients at high cardiovascular risk. Consequently, statin therapy is widely recommended as the first-line treatment of hypercholesterolemia^4^. Nevertheless, many patients with unequivocal indications for cholesterol-lowering therapy are not taking statins^5–8^.

Absence of statin therapy could be due to several phenomena: (a) discontinuation of previously initiated statin therapy (e.g. due to adverse reactions or non-adherence); (b) non-initiation of statin therapy by healthcare providers (e.g. due to therapeutic inertia); or (c) non-acceptance of providers’ statin therapy recommendations by patients. While there is extensive literature on the contributions of therapeutic inertia and non-adherence to the absence of statin therapy^9^, less is known about non-acceptance of statin therapy by patients—a situation where a patient declines to start statin therapy against their clinician’s advice.

Recent studies have shown that patients’ non-acceptance of clinicians’ statin therapy recommendations may be a significant contributor to under-utilization of statins^6,10^. However, information about the reasons for this apparently widespread phenomenon remains limited. We therefore conducted a study of patients at high cardiovascular risk who did not accept their clinicians’ statin therapy recommendations to identify the most common reasons for statin therapy non-acceptance and their relationship to the patients’ characteristics.

## Methods

### Study design

We conducted a retrospective cohort study of patients with guideline-based indications for cholesterol-lowering therapy who did not accept their clinicians’ recommendations for treatment with a statin. This study analyzed the relationships between patients’ medical history and the reasons for non-acceptance of statin therapy.

### Study population

Our study included adults (age ≥ 18) with ASCVD, diabetes mellitus (DM) or LDL-C ≥ 190 mg/dL who did not accept statin therapy recommended by clinicians at Mass General Brigham, an integrated healthcare delivery system in Eastern Massachusetts between January 1, 2000, and December 31, 2018. From this initial population, a subset of patients was randomly selected for further manual review of their medical records (to identify their reasons for statin non-acceptance) and served as the analytical population in the current study. This study was approved by the Mass General Brigham institutional review board and the requirement for written informed consent was waived (protocol # 2023P000326). This study was conducted in accordance with the ethical principles of the Declaration of Helsinki.

### Study measurements

Patients’ demographic characteristics, past medical history, laboratory tests and medications were obtained from their electronic health records (EHR). Reasons for statin non-acceptance were identified based on manual review of the patients’ clinical charts. Reasons for statin-non acceptance were classified in the following categories (chosen post hoc, based on the review of the study data): (a) preference for lifestyle modifications (i.e. an intent to use diet and / or exercise to lower cholesterol); (b) general aversion to medications (i.e. preference not to take medications, without planning to use any other approach to lower cholesterol); (c) polypharmacy burden; (d) family preference; (e) fear of adverse reactions; (f) financial and insurance barriers; (g) disbelief of statin benefit; (h) preference for alternative therapies (i.e. an intent to use non-prescription treatments, such as herbal preparations or other supplements, to lower cholesterol); and (i) pregnancy/breastfeeding. Patients could have one or more reasons from these categories. The number of medications patients were currently taking on the date of statin non-acceptance, including prescription/over-the-counter medications, and any herbal supplements or vitamins was also reviewed and recorded.

### Statistical analysis

Descriptive statistics were examined with respect to measures of central tendency, variability, distribution, number of observations and missing values. A multivariable logistic regression model was constructed to investigate the relationship between the current number of medications and concerns about polypharmacy burden as a reason for statin non-acceptance. The model was adjusted for patient age, year of study entry, diagnoses of coronary artery disease (CAD), cerebrovascular accident (CVA), peripheral vascular disease (PVD), and DM; LDL-C ≥ 190 mg/dL; demographic information (race/ethnicity, median household income by zip code, legal sex, marital status), health insurance type, smoking status, family history of DM and CAD, baseline LDL-C and Charlson Comorbidity Index as covariates.

Using the same covariates, we also constructed multivariable logistic regression models to study a) the relationship between the number of current medications and general aversion to medications as the reason for statin non-acceptance; and b) the relationship between the patient’s previous history of adverse drug reactions and fear of an adverse reaction to statins as the reason for statin non-acceptance. A marginal Cox proportional hazard regression model (with the same covariates) was constructed to investigate the relationship between preference of lifestyle modifications as a reason for statin non-acceptance and time to achievement of LDL-C < 100 mg/dL. SAS version 9.4 was used to perform all analyses. (SAS Institute, Cary, NC).

## Results

A total of 505 patients with recorded statin non-acceptance during clinician encounters in their EHR were selected for analysis. The median age of study patients was 60 years; 49.9% of patients had ASCVD (Table 1). The median number of current medications was 4. Among study patients, 293 (58.0%) had a reason for statin non-acceptance documented; 35 (6.9%) patients had multiple reasons documented. The most common reasons for statin non-acceptance (Table 2) were preference for lifestyle modifications, general aversion to medications and polypharmacy burden (e.g. “taking too many medications already”).

**Table 1:** Baseline characteristics of study patients.

Variable	All patients (*n* = 505)
Age (years), mean (SD)	60.4 (13)
Female, n (%)	298 (59)
Race / Ethnicity, No. (%)	
Asian	24 (4.8)
Black	36 (7.1)
White	395 (78.2)
Other^a^	50 (9.9)
Ethnicity, No. (%)	
Hispanic	16 (3.2)
Non-Hispanic	489 (96.8)
Study Entry Year^b^, mean (SD)	9.34 (4.3)
Charlson Comorbidity Index, mean (SD)	4.58 (3.9)
Baseline LDL-C, mg/dL, mean (SD)	15.2 (3.3)
Partnered^2^, n (%)	280 (55.5)
Smoker, n (%)	158 (31.3)
Median household income by zip code, $10,000, mean (SD)	7.18 (2.6)
English as the primary language, n (%)	458 (90.7)
Government Insurance, n (%)	210 (41.6)
CAD, n (%)	140 (27.7)
CVA, n (%)	58 (11.5)
PVD, n (%)	54 (10.7)
DM, n (%)	249 (49.3)
LDL-C ≥ 190 mg/dL, n (%)	176 (34.9)
Family history of DM, n (%)	133 (26.3)
Family history of ASCVD, n (%)	458 (90.67)
Previous adverse reactions to medications, mean (SD)	2.23 (2.8)

CAD: coronary artery disease; CVA: Cerebrovascular accident; LDL-C: low density lipoprotein cholesterol; PVD: peripheral vascular disease; DM: diabetes mellitus.

^a^Includes unknown.

^b^Since 2000.

**Table 2:** Reasons for Statin non-acceptance.

Reason	*N* (%^a^)
Preference for lifestyle modifications, n (%)	151 (51.5)
General aversion to medications, n (%)	56 (19.1)
Polypharmacy burden, n (%)	50 (17.1)
Fear of adverse effects, n (%)	32 (10.9)
Disbelief of statin benefits, n (%)	10 (3.4)
Pregnancy/Breastfeeding, n (%)	5 (1.7)
Family preference, n (%)	4 (1.4)
Financial and insurance barriers, n (%)	4 (1.4)
Preference for alternative therapies, n (%)	4 (1.4)

^a^of patients for whom a reason for statin non-acceptance was documented.

### Current number of medications and reasons for statin non-acceptance

**Fig. 1:**
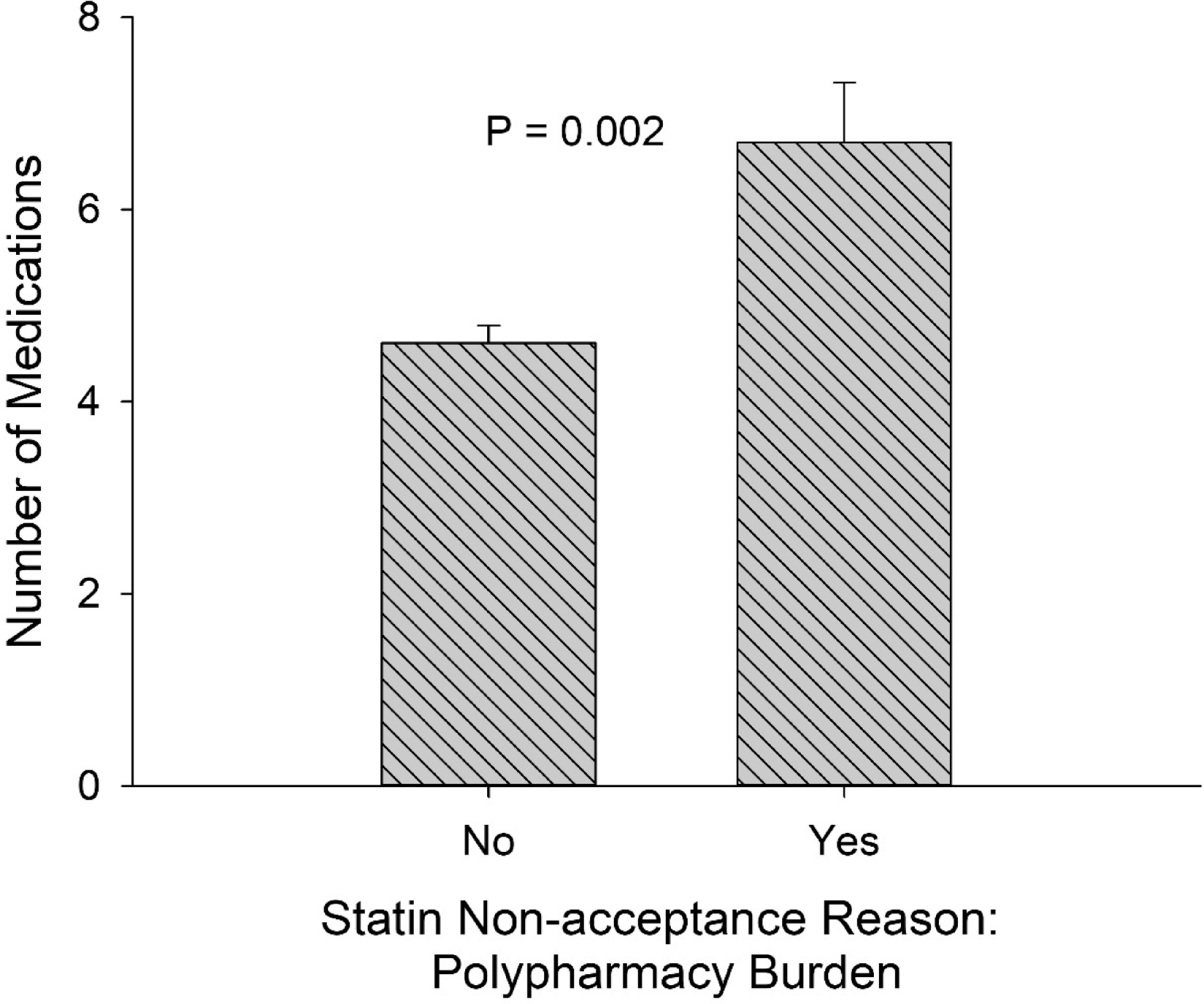
Number of medications and reasons for statin non-acceptance. Wisps indicate standard errors.

In univariate analysis, patients who reported polypharmacy burden as the reason for statin non-acceptance had a higher number of current medications (6.7 vs. 4.6, *p* = 0.002) compared to patients who did not (Fig. 1). In multivariable analysis, patients with a higher number of current medications were more likely to report polypharmacy burden as the reason for statin non-acceptance (odds ratio (OR) 1.09; 95% confidence interval (CI) 1.005–1.18, *p* = 0.037). The current number of medications was similar between patients who did vs. did not report a general aversion to medications as the reason for statin non-acceptance. (4.7 vs. 4.8, *p* = 0.76).

### History of adverse drug reactions and fear of statin side effects

**Fig. 2:**
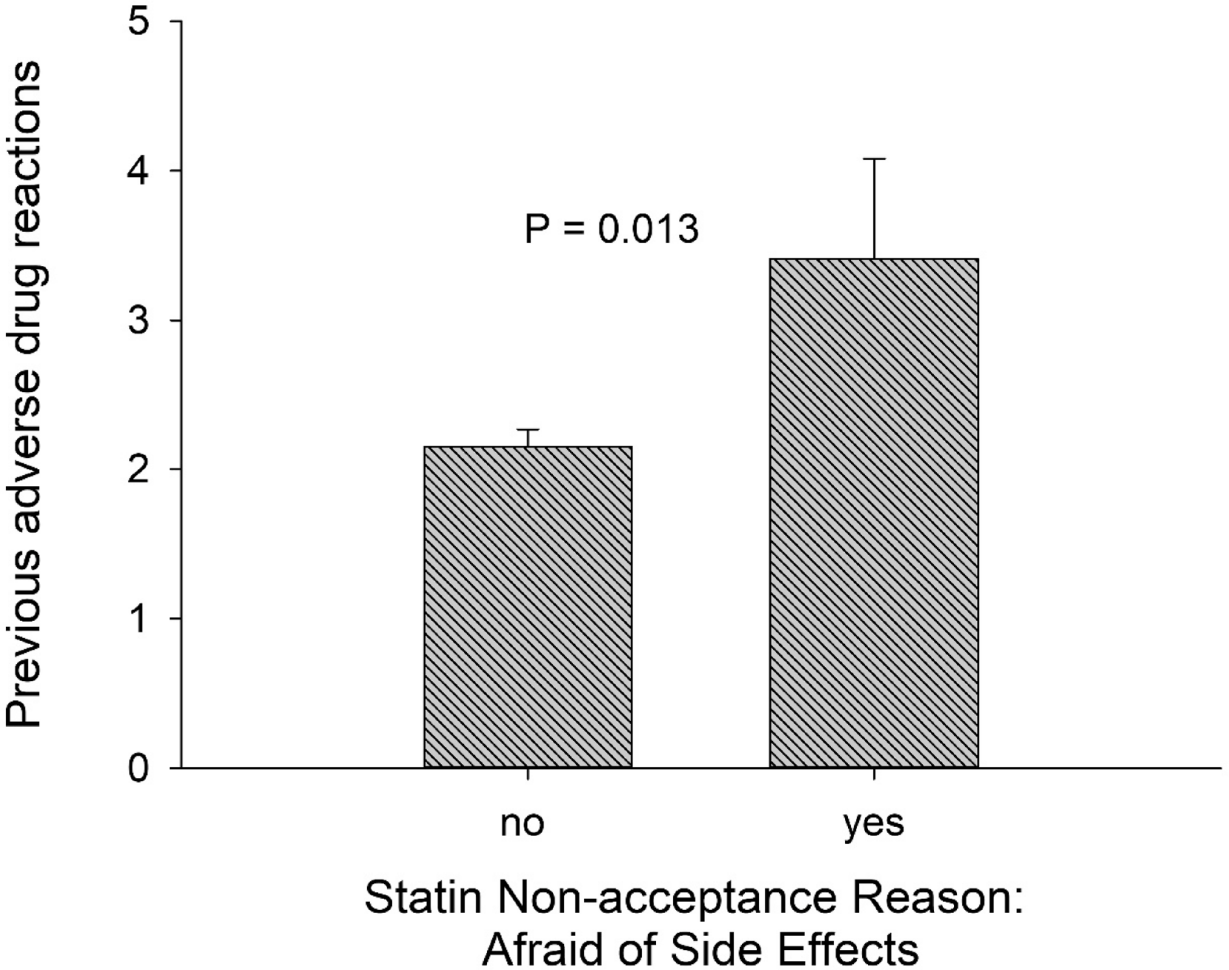
History of adverse drug reactions and reasons for statin non-acceptance.

In univariate analysis, patients who reported fear of side effects as the reason for statin non-acceptance had a higher number of previous adverse drug reactions (Fig. 2) compared to patients who did not (3.4 vs. 2.2, *p* = 0.013). In multivariable analysis adjusted for age, insurance type, smoking status, comorbidities, baseline LDL-C, and family history, each additional previously reported adverse drug reaction was associated with an odds ratio of 1.13 (95% CI 1.001–1.28, *p* = 0.048) for statin non-acceptance due to fear of statin-related adverse reactions. There was no significant relationship between the number of current medications and fear of adverse reactions as the reason for statin non-acceptance. There was no difference in prevalence of fear of statin side effects between women and men.

### Preference for lifestyle modification and LDL-C control

**Fig. 3:**
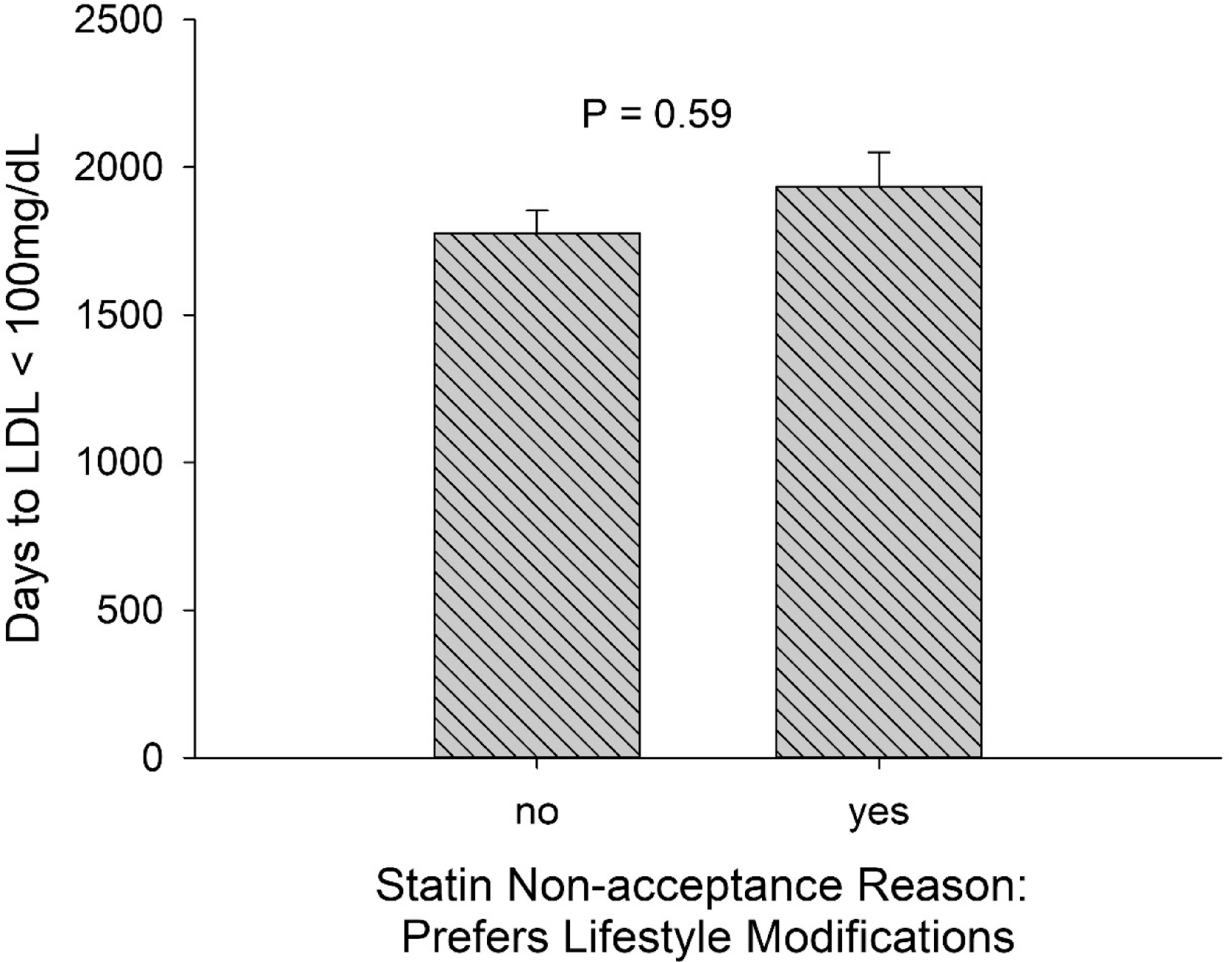
LDL cholesterol control and reasons for statin non-acceptance.

Patients who preferred lifestyle modifications over initiation of statin therapy did not have a shorter time to LDL-C control (1935 vs. 1777 days, *p* = 0.26, Fig. 3). In multivariable analysis adjusted for patients’ demographics and comorbidities, preference for lifestyle modifications as the reason for statin non-acceptance was associated with a hazard ratio of 0.998 (95% CI 0.774–1.288, *p* = 0.99) for time to LDL-C < 100 mg/dL.

## Discussion

This study identified common reasons for statin non-acceptance among individuals at high cardiovascular risk and investigated their associations with patients’ characteristics. The most frequently reported reasons for statin non-acceptance included preference for non-pharmacological interventions, concerns about taking (a large number of) medications and fear of side effects. A number of reasons for statin non-acceptance were related to the patients’ previous and current experience of non-cholesterol lowering pharmacotherapy.

Statin non-adherence and its consequences on patients’ health outcomes have been extensively studied^11^. Common factors associated with non-adherence include age, socioeconomic factors and cost, regimen complexity, smoking, social stigma and gender^12^. However, there is an important difference between statin non-adherence vs. non-acceptance: patients who are not adherent to statin therapy have already experienced it. This prior experience of statin therapy is often the driving force behind their non-adherence / discontinuation of statin therapy—in particular, the adverse reactions they might have had to statins^13,14^. For other, more expensive medications, cost—also based on the direct prior experience with the medication—can often be an important factor behind non-adherence. On the other hand, concerns about cost were not a common reason for statin non-acceptance [among patients who never tried a statin] in our study, even though it spanned a significant period of time when most statins did not have generic versions available and could have been expensive for patients. Similarly, we found that fear of adverse reactions was a relatively infrequent reason for statin non-acceptance.

A previously published investigation based on the PALM registry evaluated reasons for statin non-acceptance in a smaller sample of 153 patients^15^. This survey-based study also found that preference for lifestyle modifications or alternative therapies and general aversion to medications were common reasons for statin non-acceptance. Unlike the present analysis, the PALM registry study reported that concern about possible adverse reactions was the most common reason statin non-acceptance. This difference may have been in part due to the fact that concern about adverse reactions was the first option in the survey used in the study; effects of question / answer order on survey results are well documented^16^. Importantly, availability of extensive EHR data allowed the present study to analyze the relationships between the patients’ reasons for statin non-acceptance and a) their past medical history and b) their subsequent clinical course, allowing greater insight into risk factors and consequences of statin non-acceptance.

Another medication that has been shown to be commonly not accepted by patients is insulin^17,18^. However, the reasons for insulin non-acceptance are significantly different from statin non-acceptance. Patient surveys show that the most common reasons for non-acceptance of insulin include fear of dependence, fear of pain and perception of initiation of insulin therapy as a personal failure^19,20^. In contrast with statin therapy, few patients declining insulin therapy were concerned about potential side effects. On the other hand, similarly to individuals who did not accept statin medications, patients who declined insulin therapy often also prefer trying lifestyle modifications, believing they can make effective changes to their current diet and exercise regimens^19^. It is thus likely that reasons for medication non-acceptance can vary significantly based both on clinical characteristics and social perceptions of both the medication and the disease being treated. Clinical approaches to address medication non-acceptance should therefore be tailored accordingly.

A number of reasons for statin non-acceptance were related to the patients’ medical history and treatment. Importantly, concerns about the burden of polypharmacy were more common among patients who were taking a greater number of medications when being recommended a statin. Comorbidities requiring multiple medications, such as cardiovascular disease, hypertension and diabetes, are common among patients with hypercholesterolemia^21^. Polypharmacy burden has also been found to be associated with decreased medication adherence^22,23^ and with an increased risk for drug-drug interactions^24,25^. Strategies for reducing medication burden, such as fixed-dose medication combinations, once daily dosing and regular medication reviews could therefore potentially reduce medication non-acceptance as well as have multiple other benefits for patients’ long-term health^26–29^.

Another important aspect of patients’ medical history that was associated with non-acceptance of statin therapy recommendations was previous adverse reactions to non-cholesterol lowering medications, linked to an increased frequency of fear of adverse reactions to statins as the reason for their non-acceptance. Concerns about adverse reactions to statins also remain a major factor in poor adherence to these medications^30^. However, in randomized controlled trials, there are often minimal differences in incidence adverse drug reactions between statin and placebo groups^31,32^. Additionally, observational studies have shown that many patients who report adverse reactions to statins are subsequently able to resume statin therapy^13,33^. Mitigation strategies aimed at reducing patients’ concerns about statin adverse reactions, such as patient education and lower-intensity statin dosing regimens, could therefore play an important role in increasing patients’ acceptance of statin therapy^34^.

Preference for lifestyle modifications was by far the most common reason for statin non-acceptance by patients in our study. However, studies show that even intensive lifestyle lipid-lowering interventions lead only to modest reductions in LDL-C and do not decrease the incidence of cardiovascular events^35,36^. Consistent with these findings, opting for lifestyle modifications was not associated with a shorter time to LDL-C control in our study. Consequently, patients at high cardiovascular risk who choose lifestyle modifications over statin therapy may benefit from close follow-up and re-addressing the need for adding statin therapy to their diet/exercise regimen if LDL-C lowering goals are not met.

Another barrier to acceptance of statin-therapy may be the widespread social stigma and skepticism toward statin medications. Countless books, mainstream social media outlets and search engines often propagate misinformation about statins and exaggerate potential adverse reactions^37,38^. Common controversies surrounding statins include claims that these medications are falsely advertised by pharmaceutical companies for monetary purposes^39,40^. These platforms frequently recommend inappropriate alternatives or home remedies. Addressing existing misinformation on the internet is therefore crucial to solving the problem of statin non-acceptance (though admittedly a challenge).

This study has a number of strengths. The analysis focused on patients at high cardiovascular risk—a population for whom optimization of clinical approach to cholesterol-lowering therapy is most urgent. Initial data collection was conducted through careful manual record review, allowing thorough extraction of information. We comprehensively analyzed encounters between patients and primary care physicians documented in EHRs. Unlike in a prospective study, informed consent was not required from patients prior to participation. Patients who are reluctant to start statin medications may also be hesitant to enroll in such studies, resulting in a selection bias that has been well described in published research^41,42^. The present study therefore provides unfiltered insights into patients’ reasons for statin non-acceptance through real-world evidence.

Our study also had several limitations. First, its sample size is limited, possibly impacting proper statistical assessment of relationships between patient characteristics and reasons for statin non-acceptance. Some of the data in the analysis may be missing due to incomplete documentation in the EHR. In particular, a large fraction of study patients did not have a reason for statin non-acceptance documented in their EHR records. If the distribution of the reasons for statin non-acceptance was different among the patients for whom it was not documented, the study findings may have been biased. The study did not include patients with lower cardiovascular risk; their reasons for non-acceptance of statin therapy recommendations may be different than described in this report. The analysis of LDL_-_C levels after choosing lifestyle modifications did not include information on whether these patients subsequently initiated a statin or another cholesterol-lowering medication. This study was conducted at a single site in Massachusetts, which may have affected its generalizability to other healthcare settings. Lastly, the retrospective design of this study may yield biases and confounded results. More prospective, multi-center studies in the future are needed to complement and confirm our conclusions.

## Conclusions

Patients’ reasons for non-acceptance of statin therapy represent a distinct set of preferences and concerns, many of which are rooted in their past and present medical experience. Importantly, patients’ preference for lifestyle modification did not appear to translate into decrease in LDL-C. Appropriately addressing patients’ concerns as well as close follow-up and monitoring of the effectiveness of alternative therapeutic approaches are important to maximize cardiovascular risk reduction in individuals not ready to initiate statin therapy.

## Data Availability

Raw patient-level data used in this study cannot be shared openly to protect patient privacy, based on the Mass General Brigham institutional policies. De-identified patient-level data are available under a Data Use Agreement (DUA), in accordance with the Mass General Brigham institutional policies. Data requests can be made to the Mass General Brigham Institutional Review Board at irb@mgb.org.
